# Drinking Songs: Alcohol Effects on Learned Song of Zebra Finches

**DOI:** 10.1371/journal.pone.0115427

**Published:** 2014-12-23

**Authors:** Christopher R. Olson, Devin C. Owen, Andrey E. Ryabinin, Claudio V. Mello

**Affiliations:** 1 Department of Behavioral Neuroscience, Oregon Health & Science University, 3181 SW Sam Jackson Park Road L470, Portland, Oregon, United States of America 97239–3098; 2 Psychology Department, Lewis and Clark College, 0615 S.W. Palatine Hill Road, Portland, Oregon 97219, United States of America; UCLA, United States of America

## Abstract

Speech impairment is one of the most intriguing and least understood effects of alcohol on cognitive function, largely due to the lack of data on alcohol effects on vocalizations in the context of an appropriate experimental model organism. Zebra finches, a representative songbird and a premier model for understanding the neurobiology of vocal production and learning, learn song in a manner analogous to how humans learn speech. Here we show that when allowed access, finches readily drink alcohol, increase their blood ethanol concentrations (BEC) significantly, and sing a song with altered acoustic structure. The most pronounced effects were decreased amplitude and increased entropy, the latter likely reflecting a disruption in the birds’ ability to maintain the spectral structure of song under alcohol. Furthermore, specific syllables, which have distinct acoustic structures, were differentially influenced by alcohol, likely reflecting a diversity in the neural mechanisms required for their production. Remarkably, these effects on vocalizations occurred without overt effects on general behavioral measures, and importantly, they occurred within a range of BEC that can be considered risky for humans. Our results suggest that the variable effects of alcohol on finch song reflect differential alcohol sensitivity of the brain circuitry elements that control different aspects of song production. They also point to finches as an informative model for understanding how alcohol affects the neuronal circuits that control the production of learned motor behaviors.

## Introduction

Alcohol consumption has wide-ranging effects on behavioral and cognitive functions, including verbal and non-verbal retention [1], and can lead to structural brain changes [2], [3]. Particularly intriguing are the effects on human speech, with alcohol exposure leading to markedly altered and degraded vocalizations [4], [5]. Indeed, measurements of altered speech have been proposed as diagnostic of an inebriated state [6], [7]. While many of the major effects of alcohol in humans have been replicated in rodent [8] and non-human primate [9] models, yielding insights into how alcohol disrupts cognitive traits and their underlying neuronal mechanisms, the alcohol impairment of human speech production remains poorly understood. This is due in part to the intrinsic difficulties in performing detailed mechanistic studies in humans, but also because an appropriate animal model has not yet been developed for investigating speech-related deficits due to alcohol.

To investigate how alcohol affects a behavioral trait with significant analogies with human speech, we used the zebra finch, a representative songbird species, and a powerful model for the study of mechanisms that underlie vocal learning and production. Vocal learning is a vital prerequisite for human language acquisition, but is rare among animals, described only in cetaceans, bats, and broadly among three avian lineages, namely songbirds, hummingbirds, and parrots [10], [11]. Most of these mammalian species do not easily lend themselves to mechanistic studies of brain function in laboratory settings, and traditional model organisms like rodents and non-human primates lack vocal learning and associated brain circuits [12]. Importantly, male zebra finches are highly motivated to sing under a variety of conditions, and there are remarkable analogies in how zebra finch song and human speech are learned and produced [10], [13]. Both groups require a prolonged development period with proper environmental cues and specialized brain circuitry for vocal acquisition. In humans, this process depends on exposure to vocalizations by parents or peers [14] and on auditory-vocal motor feedback acting on brain regions associated with verbal and vocal-motor processing [15]–[17], resulting in neural encoding that is required for the consolidation of learned vocal patterns. Similarly, juvenile zebra finches require exposure to tutor song to form an auditory memory, and learn to imitate that song over a prolonged period of vocal practice that relies on auditory feedback [13], [18]. Importantly, a set of sophisticated tools and algorithms are currently available for quantitative analyses of bioacoustics features of finch song [19].

**Figure 1:**
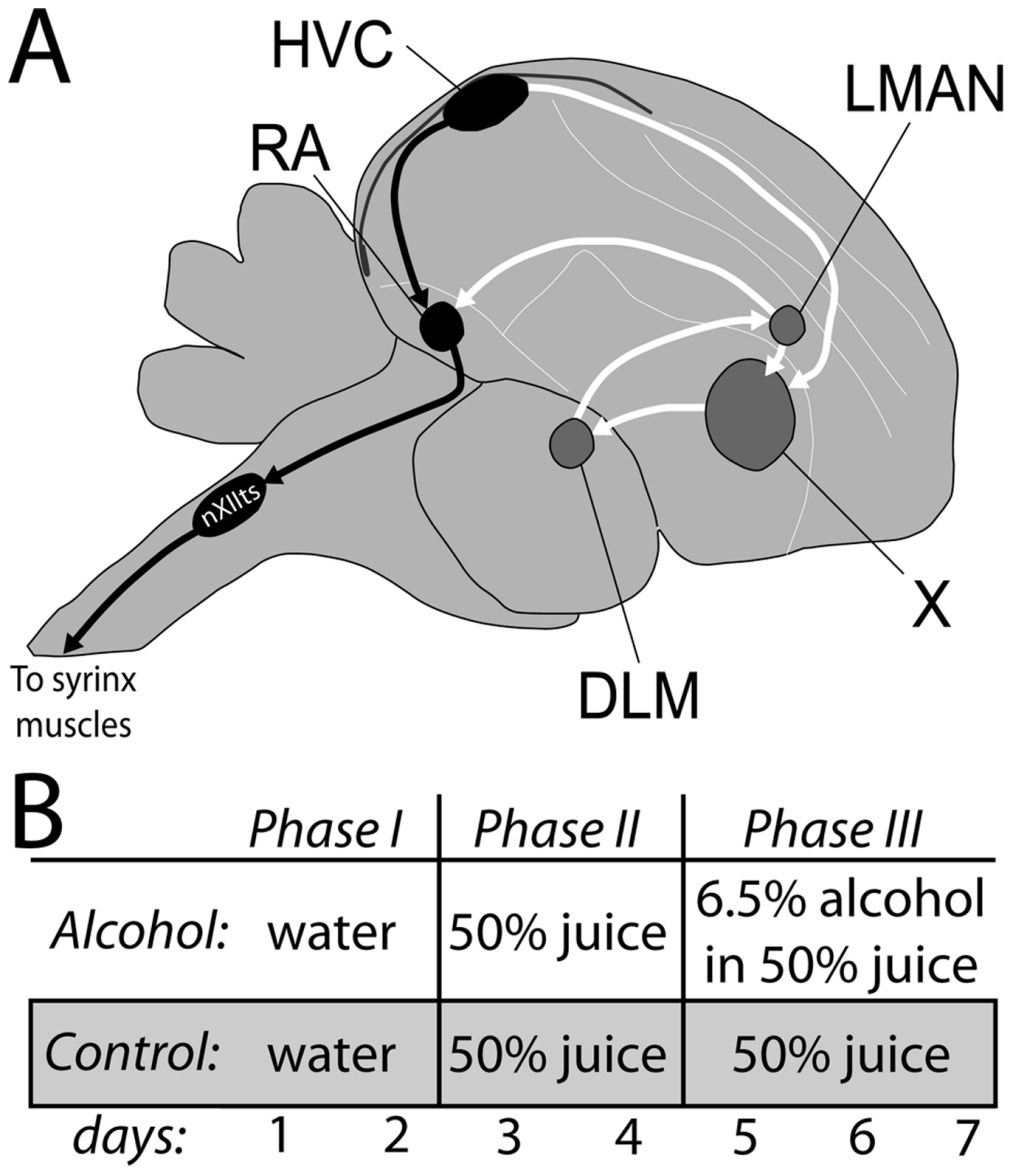
The finch song system and experimental paradigm. (A) The neuronal song system consists of a posterior vocal motor pathway (black): pre-motor cortical nucleus HVC projects to motor nucleus RA (robustus of the arcopallium), which projects to brainstem medullary nXIIts respiratory and vocal (syrinx) motor neurons, and the anterior pathway (white): HVC (a proper name), which projects to striatal Area X (X), and from there, sequentially to thalamic nucleus DLM (dorso-lateral division of the medial thalamus), to cortical nucleus LMAN (lateral magnocellular nucleus of the nidopallium) and to RA. (B) The study design assigned adult male finches to alcohol or control drinking treatments. They were then taken through three sequential Phases where they were provided with water (Phase I), 50% juice (Phase II), and alcohol in juice or juice only (Phase III).

The vocal control circuitry of humans and songbirds also have remarkable similarities: while human vocal production is known to depend on cortical regions, the production and learning of birdsong requires a set of cortical-like and basal ganglia structures, whose primary output is onto vocal and respiratory neurons in the brainstem (Fig. 1A). Of note, nucleus RA occupies a position in this pathway analogous to the layer 5 motor neurons within the oral-motor and laryngeal representation areas of the human primary motor cortex, which project onto brainstem laryngeal centers for vocal control [10], [20], allowing for cortical control of the production of refined acoustic (spectral and temporal) features that are characteristic of learned vocalizations. Vocal learning in finches and humans also share important molecular underpinnings: both organisms require an intact FOXP2 gene for the proper learning of vocal patterns [21]–[23]. Thus finches stand out in their applicability to understanding how complex motor abilities like learned vocalizations are affected by alcohol.

We show here that zebra finches will consume alcohol when it is provided to them, resulting in elevated blood ethanol content (BEC). While alcohol exposure does not visibly affect general behaviors, willingness or motivation to sing, or variability of vocal output, it has marked effects on acoustic features of learned song, particularly entropy and amplitude. Thus, our study points to a songbird species as an informative model organism for further mechanistic studies on the cognitive actions of alcohol. Based on the organization of the song control circuitry, we suggest that some of the major effects of alcohol on vocalizations are exerted at the level of cortical vocal pathways.

## Results

### General effects of exposure to alcohol

**Figure 2:**
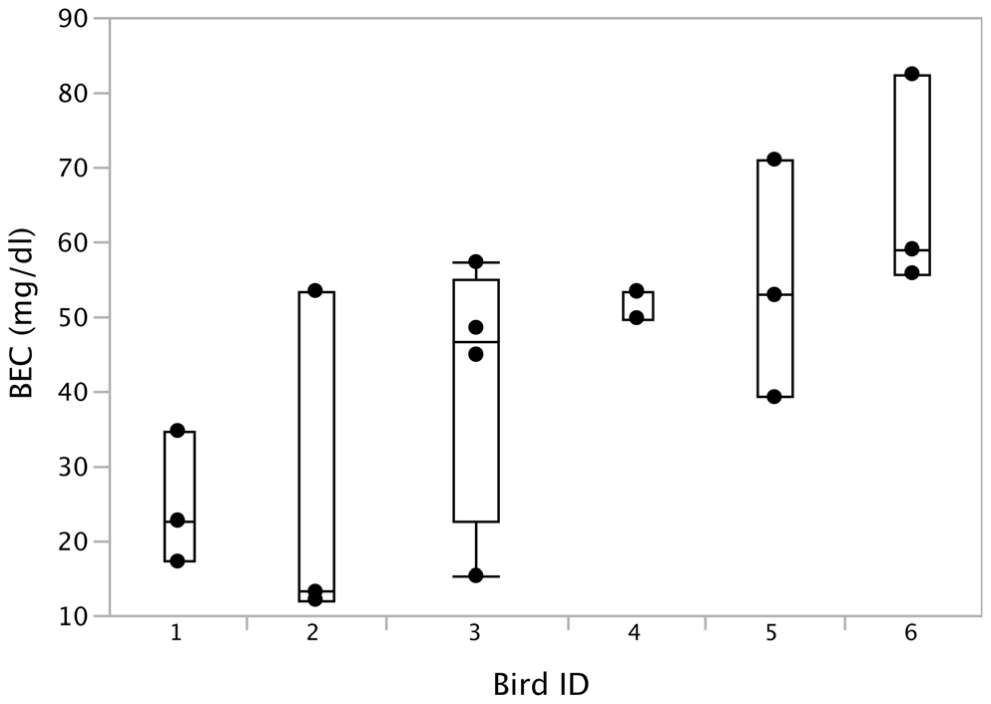
Blood ethanol concentrations (BEC) in male zebra finches. Birds were provided with 6.5% alcohol in 50% juice during Phase III of our experimental paradigm.

Our basic paradigm (Fig. 1B) consisted of recording female-directed song while sequentially providing birds with water (Phase I), juice (Phase II), and alcohol plus juice vs juice only (alcohol vs. control groups; Phase III) over several consecutive days. Fluid intake did not differ between control and experimental birds during phases I and II, but the experimental birds increased their fluid consumption by ∼50% between phases II and III, illustrated by an interaction effect between group and phase (S1 Fig., repeated measures ANOVA F_1, 10_ = 5.4, p = 0.042). The experimental birds at Phase III consumed a daily ethanol dose of 16.4±2.6 (s.e.) g/kg, or ∼0.26ml per bird per day. All birds that were provided alcohol in Phase III showed detectable BECs (mean 44.0 mg/dl), but with considerable variability among individuals (One-way ANOVA F_5, 18_ = 3.26, p = 0.04; Fig. 2). BEC ranged from undetectable on some days for some birds, to a maximum of 82.4 mg/dl for one bird. The relationship between daily dose and BEC was not significant (F_1, 22_ = 1.75, p = 0.20), indicating that the finches could have varied their fluid intake throughout the day, or metabolized alcohol at a very high rate.

To examine the latter possibility, we conducted a small scale metabolic clearance study, akin to a paradigm commonly used to study alcohol clearance rates in rodents [24]. Zebra finches received injections of two different doses of alcohol, and BECs were determined at increasing intervals after the injection. The results show a high BEC within 30 minutes of the injection, followed by a very gradual decline, such that 3 hours later the birds had metabolized only ∼25% of the original dose (S2 Fig.). This contrasts with existing data for C57BL/6 mice, where nearly all alcohol has been removed within 3 hours after the injection (2 g/kg). Thus, finches appear to have a slow metabolic clearance of alcohol, and are likely to sustain relatively stable BEC for periods of hours.

We did not detect visible effects on the birds’ general behaviors or health, as indicated by the normal appearance of feathers and the ability to perch, feed, maintain normal posture and fly inside the cage. We also did not observe overt signs of stress in either alcohol or control groups, including postural changes, drooped wings or puffed feathers, or changes in behavior, including inactivity, panting, closed eyes or non-responsiveness towards other finches. Alcohol did not affect the birds’ use of perches when females were presented, and birds appeared to sing from the same position in the chamber throughout all phases of the study. Body mass also remained constant across phases for the birds that were provided alcohol (F_2, 34_ = 1.03, p = 0.37). Importantly, all birds maintained their ability to sing, and produced zebra finch-typical song; even though the birds varied in the amounts of song produced, as normally happens for finches, enough recording data were obtained for quantitative analysis. No detectable BECs were seen in control birds that did not receive alcohol in Phase III.

### Effects of alcohol on singing behavior

**Figure 3:**
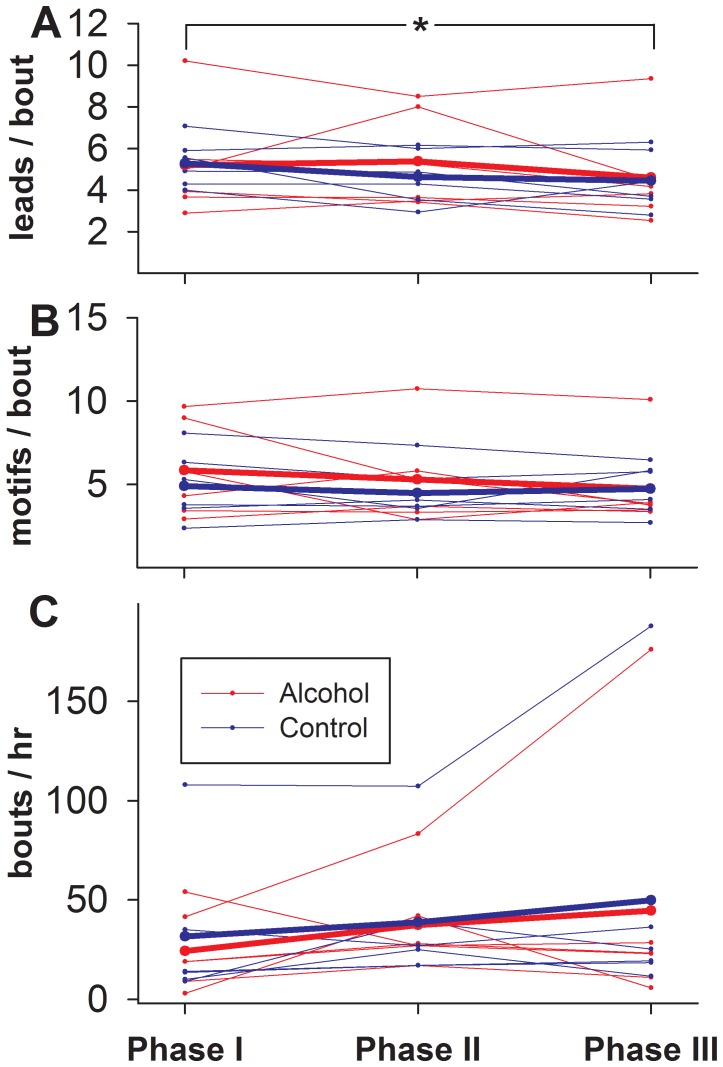
Effect of alcohol drinking on singing behavior. Plotted are the individual and treatment group means of (A) number of lead notes per bout, (B) the mean number of motifs within a bout and (C) the mean number of bouts per hour across Phases for birds that received alcohol (red) in Phase III and for controls (blue). Small symbols connected by dotted lines represent individual bird means, while large symbols connected by solid lines represent the treatment groups. An * indicates a significant change across phases for both the control and alcohol treatments, combined.

To assess whether alcohol affects the motivation to sing, we analyzed whether it affected the number of bouts sang, the numbers of motifs within each bout, or the number of lead notes prior to each bout during the 60 minutes of recording following the introduction of the target female to the recording chamber. We used repeated measures models to test for effects of alcohol on these song variables, separately comparing groups (alcohol vs. controls) and experimental Phase. We note that both the alcohol and control groups sang robustly on all days, with no significant group differences in the levels of singing (Fig. 3; Table S1 in S1 File). Interestingly, we detected a phase effect on the number of lead notes produced, with a decline over the course of the experiment (Fig. 3A; F_2, 8_ = 1.13, p<0.05). Yet the number of lead notes did not differ between the alcohol and control groups (F_1, 9_ = 0.0004, p = 0.95). Since the presence of multiple lead notes in zebra finch song is more typically associated with female-directed than undirected song [25], it can be considered an indicator of the motivation of males to sing to females. We therefore interpret the decline in the number of lead notes seen in both groups as a small decline in the motivation to sing to females after prolonged exposure to same housing conditions, regardless of the presence of alcohol.

We next tested whether BEC correlated with any of the song measures, and found no significant effects of BEC level on the mean number of motifs (p = 0.53) or lead notes (p = 0.73) associated with each bout (individual birds varied greatly, p<0.0001) and no interactions (p = 0.66 and 0.92, respectively). When testing the BEC level against the number of song bouts per hour, there was a significant bird by BEC interaction (p = 0.001); examination of the data scatter revealed that one of the birds deviated markedly from the others, its singing rate increasing with high BECs (S3 Fig.). Repeating the analysis with this individual omitted made the interaction disappear (p = 0.47), and the number of songs decrease with BEC (p = 0.029), indicating that a typical finch response to high BEC is more likely to be a small decrease in song rate.

### Effects of alcohol on acoustic features of songs

**Figure 4:**
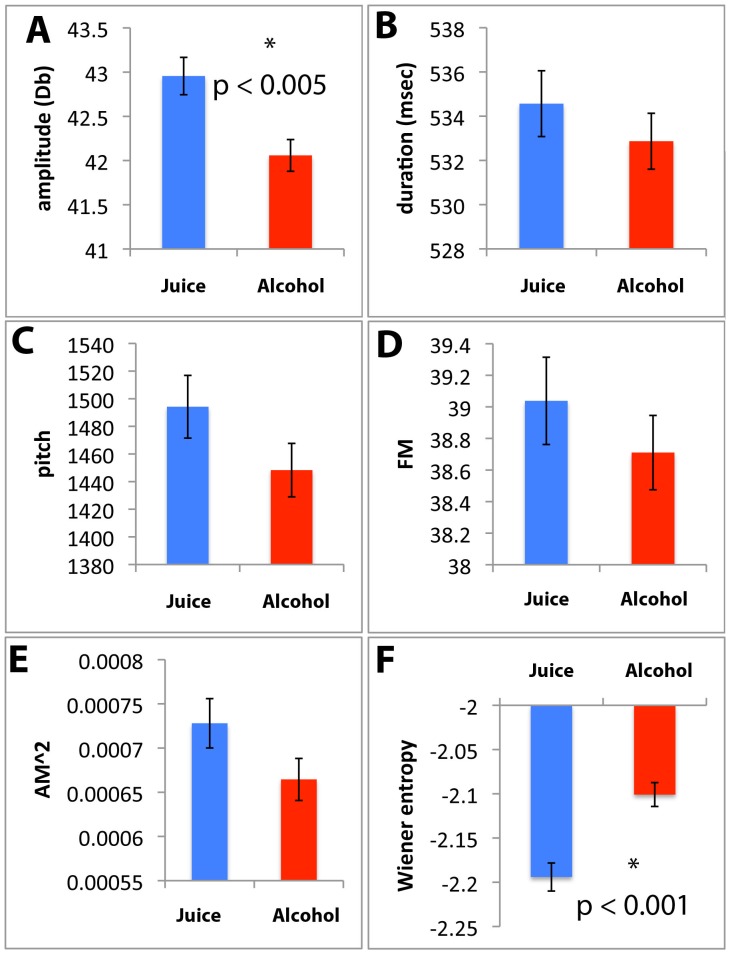
Effects of alcohol drinking on song acoustic features. Plotted are the values of least-square means of each acoustic feature (A–F) from whole-motif measurements of all individuals; error bars are standard errors of the means. * indicates a significant difference.

Initially we tested for the effect of alcohol on four song spectral features (pitch, frequency modulation (FM), amplitude modulation (AM), and Wiener entropy) as well as amplitude averaged over the duration of whole motifs, and motif duration. We used a MANOVA to test for effects when comparing songs recorded in Phases II and III, while also controlling for variation in individual birds. For birds that were provided with alcohol during Phase III, there were strong individual effects (Wilk’s λ_6, 36_ = 2.12×10^9^, p<0.0001) and a detectable difference in the multiple variables analyzed between Phases II and III (F_6, 17_ = 1.04, p = 0.0375). For the control birds, however, no significant differences were found between Phases II and III in the multiple acoustic variables analyzed (F_6, 19_ = 0.74, p = 0.0715), despite similarly large differences among individuals (Wilk’s λ_6, 36_ = 26.759×10^10^, p<0.0001). A *post hoc* analysis of the birds that received alcohol in Phase III showed that amplitude significantly decreased (p<0.0001) and entropy increased with alcohol (p<0.01), whereas other song features did not change (Fig. 4; Table S2 in S1 File), even though there were trends suggesting declines in several features. Control birds showed no significant changes in any spectral feature over the 7-day recording period (Table S2 in S1 File), excluding the possibility that the changes in amplitude and entropy in the alcohol group result from non-specific factors, such as stress related to the extended stay in the recording box.

**Figure 5:**
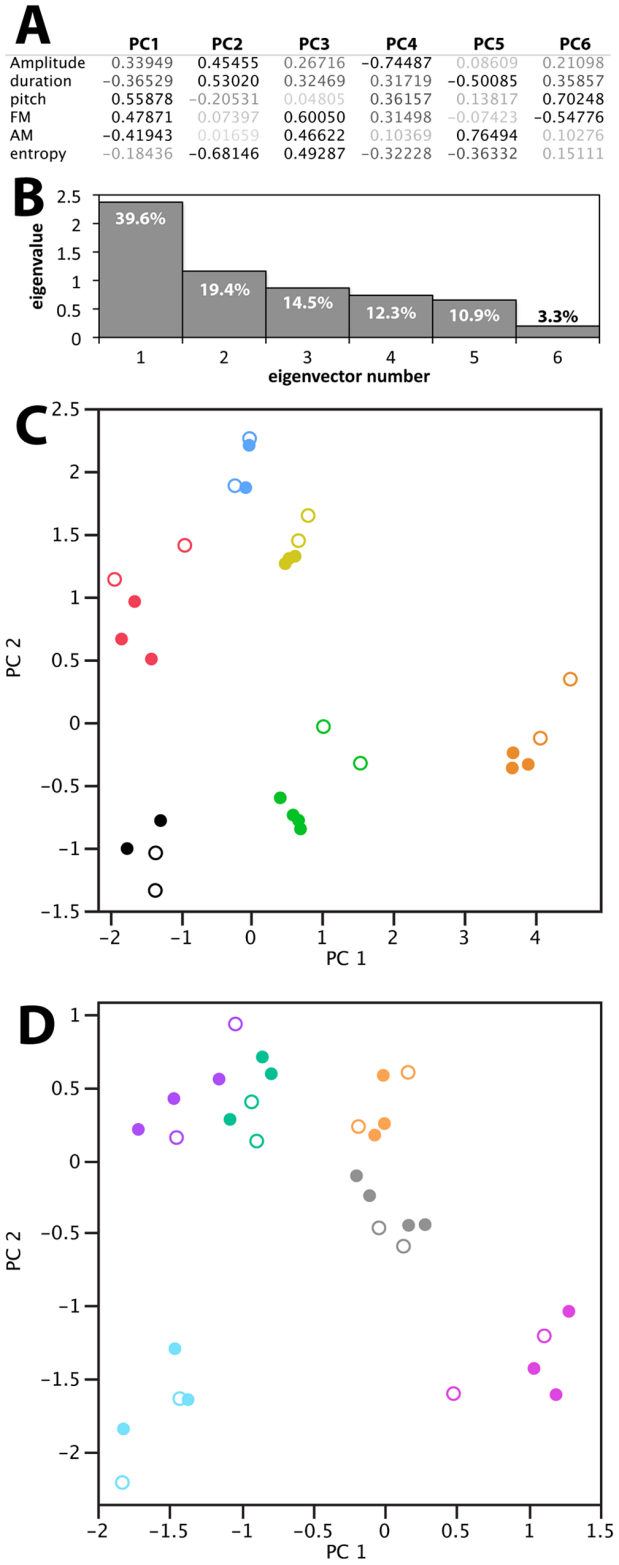
Principal Component Analysis (PCA) of the effects of alcohol drinking on acoustic parameters of whole song motifs. A. Relative contributions of different acoustic parameters to PCA eigenvectors; the darkness of the print indicates the strength of contribution. B. Scree plot indicating the percent contribution by each eigenvector to the total variation along the axis. Plots of PC2 vs. PC1 for (C) the Alcohol treatment and (D) the Control treatment: unique colors denote individual birds; open and solid circles indicate daily values recorded in Phase II (no alcohol) and Phase III (alcohol) respectively; some individual values are not visible as they overlap.

To better understand the effects of alcohol on song acoustic features we subjected the measurements obtained over entire songs from the Alcohol and Control groups (including Phases I/II and III) to Principal Component Analysis (PCA). The correlation matrix that parses the major axes of variation in the data indicate that the first eigenvector (PC1; ∼40% of the variation) explains primarily the non-entropy spectral features of pitch, FM and AM, whereas the second eigenvector, (PC2; ∼20% of the variation) is composed primarily of entropy and to a lesser degree motif duration and amplitude (Fig. 5A,B; note the opposite signs for entropy vs. amplitude and duration). Thus, the six acoustic features that we measured can be conveniently described in terms of two condensed eigenvectors. When plotting PC2 vs. PC1 to create an acoustic space for the alcohol treatment (Fig. 4C), we noted that the shifts between the Phase I/II and Phase III (alcohol) conditions occurred along both PC1 and PC2 axes with shifts down and to the left. For most birds the shifts were consistently towards higher values, likely a consequence of higher entropy and lower amplitude values under alcohol. One bird with no shift along either axis (Fig. 5C, blue) also had the lowest BECs among all subjects in the study (from undetectable to 34.7 mg/dl). Another bird showed a shift in the opposite direction, indicating a increase in PC2 (Fig. 5C, black). This bird had the least amount of singing (2 to 17 bouts per day), regardless of Phase, and its recordings had numerous female calls overlapping the male’s song. This resulted in a much limited dataset for acoustic analysis, suggesting that this bird was under-sampled compared to the others. No shifts in the acoustic space were observed when the same analysis was performed for the control group (Fig. 5D). Overall, our data show that these multiple acoustic features can be reduced to one or two primary axes, and that alcohol has significant global effects on some song spectral features, decreasing amplitude and increasing entropy.

**Figure 6:**
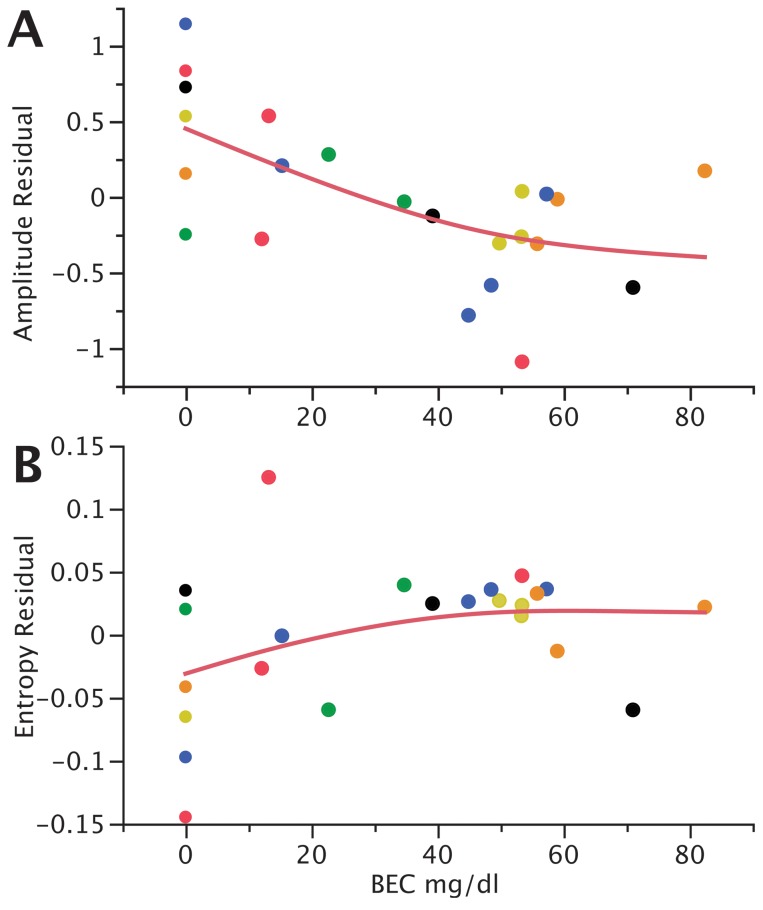
Dose-relationships between BEC and motif-level (A) amplitude and (B) entropy. The vertical axis is centered on residual values calculated around individual birds, and individual birds are represented by unique colors. The traces are spline fits (λ = 100,000).

We next examined how amplitude and entropy, the two variables for which we detected significant effects of alcohol, changed across the range of BECs measured. We found that the values decreased with BEC for amplitude (t = −4.53, p = 0.0001; Fig. 6A) and increased for entropy (t = 2.15, p = 0.04; Fig. 6B); the effects were most obvious in the lower range of BECs, up to ∼40 mg/dl.

### Effects of alcohol on spectral features of song syllables

**Figure 7:**
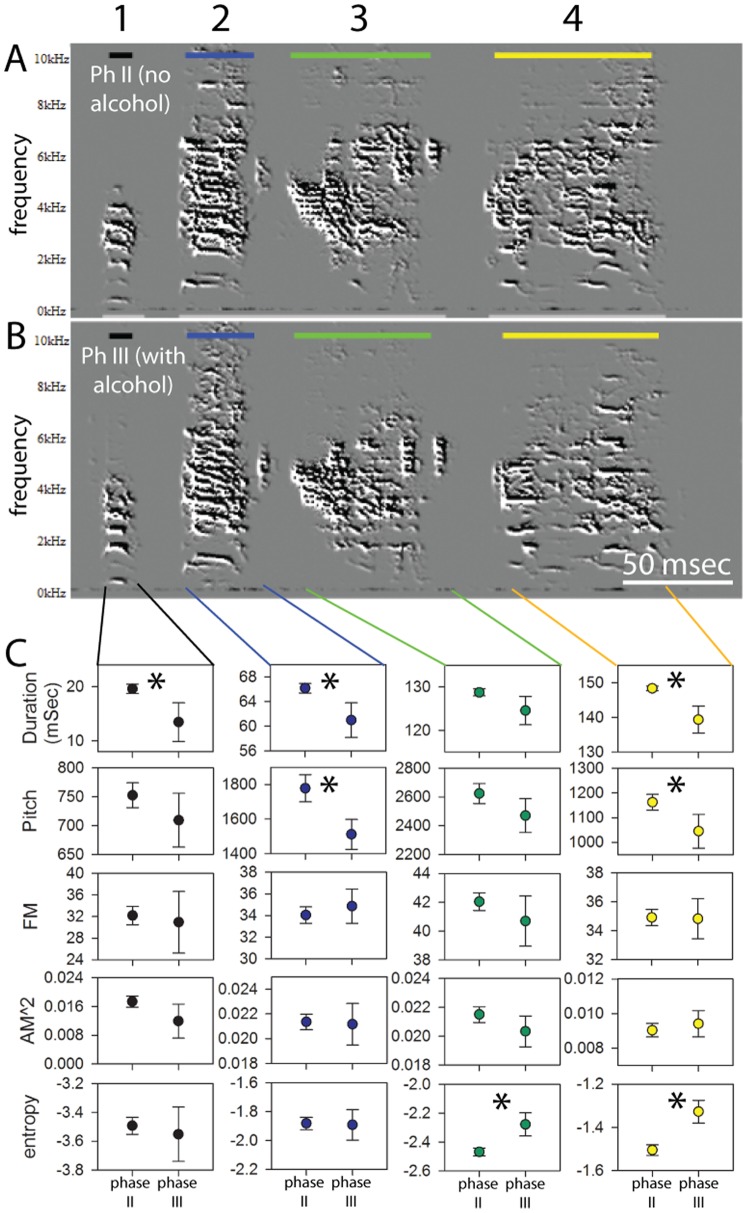
Effects of alcohol drinking on individual song syllables. Shown are sample spectrograms of single motifs from the same bird in the alcohol group, recorded during (A) Phase II (no alcohol) and (B) Phase III (alcohol); individual syllables are color-coded and labeled 1–4. (C) Plotted are mean values of acoustic features of each syllable during Phases II and III; error bars are Bonferroni-corrected 95% confidence intervals, * indicates significant differences between no alcohol and alcohol conditions.

The various syllables that make up a zebra finch song motif differ greatly in acoustic structure, reflecting a diversity in the neural encoding and vocal production mechanisms the birds use for generating them. We therefore wondered whether alcohol may exert differential effects on different syllables. To address this possibility, we conducted an analysis of spectro-temporal features in repeated measures comparisons of multiple vocal renditions with or without alcohol, but now using single syllables instead of whole motifs as analyzed units. To the human ear the differences in amplitude in individual syllables between Phases II and III were apparent and there were noticeable differences in the spectral quality of sounds when the speed of recordings was slowed down (See S2 File for an audio-video presentation of songs and spectrograms). With quantitative acoustic analysis we detected significant effects, but close examination revealed that, as predicted, not all syllables were equally affected (Fig. 7 shows a representative example of this analysis). Different syllables showed effects on different acoustic parameters, while several other syllables showed no effects in any parameters. Consistent with the whole motif analysis, of 21 syllables analyzed, 11 increased entropy, but other syllables also had detectable changes in parameters for which no effects were seen when analyzing across the whole motifs. This included changes in duration (n = 2 increases and 14 decreases), decreases in pitch (n = 8), decreases in FM (n = 5), and decreases in AM (n = 10), in varying combinations for different syllables. Duration was the only parameter that showed changes in both directions, while the other spectral features changed either in one direction or showed no change. We also tested how acoustic features of individual syllables may change across the range of BEC values that we recorded on different days. From the song recordings we obtained we first calculated the mean acoustic features for each syllable for each day with a given BEC. Using a model where syllables were nested within bird ID as random factors, and BEC was included as the main factor, we found that amplitude and syllable duration both decreased and that entropy increased with increased BEC (Table 1, S4 Fig.). In contrast, although some individual syllables were noted to change in terms of pitch, FM or AM, these acoustic features did not reliably change with increased values of BEC. We next tested whether the fraction of acoustic features that changed from the control values increased with increases in BEC. Here BEC did not have a measurable effect on the fraction of responding acoustic features in the model (F_1, 48_ = 2.5, p = 0.12). However, we again noted strong differences in the fraction of responding acoustic features among syllables (F_17, 48_ = 3.1, p = 0.001), in line with our observations that alcohol alters some, but not all, of the acoustic features of individual syllables within a bird’s motif (e. g. see Fig. 7). There was also an individual bird effect (F_5, 48_ = 3.5, p = 0.02), where some birds had more acoustic features altered than others.

**Table 1:** ANOVA tables for the effects of BEC (a main effect) and individual Bird ID and syllable nested within bird ID as random effects.

Acoustic Feature	Model effects	DF	F	p	Direction of change
Amplitude	**BEC**	**1, 65**	**41.3724**	<.0001*	**Decrease**
	Bird ID	5, 65	6.5446	0.0020*	
	Syllable [Bird ID]	15, 65	111.8904	<.0001*	
Duration	**BEC**	**1, 65**	**5.6347**	0.0206*	**Decrease**
	Bird ID	5, 65	0.2991	0.9058	
	Syllable [Bird ID]	15, 65	1502.681	<.0001*	
Pitch	**BEC**	**1, 65**	**0.0020**	**0.9647**	**No change**
	Bird ID	5, 65	1.1221	0.3904	
	Syllable [Bird ID]	15, 65	222.0660	<.0001*	
FM	**BEC**	**1, 65**	**0.0010**	**0.9751**	**No change**
	Bird ID	5, 65	0.5750	0.7184	
	Syllable [Bird ID]	15, 65	206.4407	<.0001*	
AM	**BEC**	**1, 65**	**0.7012**	**0.4055**	**No change**
	Bird ID	5, 65	0.8497	0.5360	
	Syllable [Bird ID]	15, 65	152.0767	<.0001*	
Entropy	**BEC**	**1, 65**	**4.2399**	0.0435*	**Increase**
	Bird ID	5, 65	0.6701	0.6522	
	Syllable [Bird ID]	15, 65	208.5539	<.0001*	

**Figure 8:**
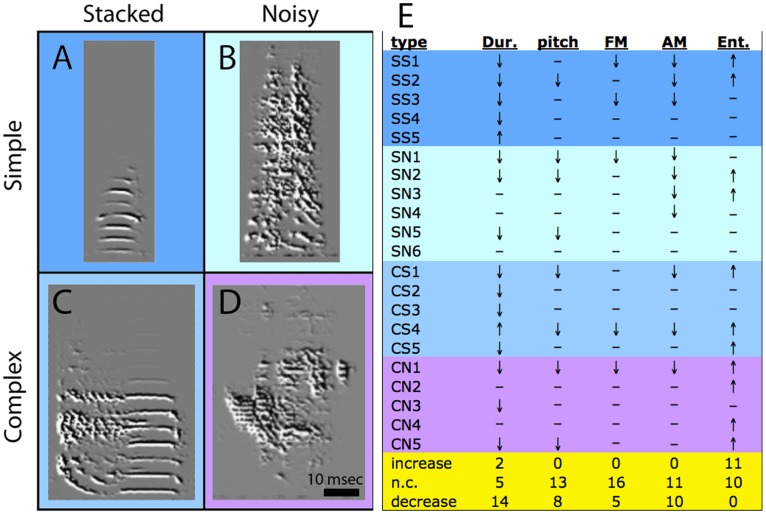
Left: Syllable types in zebra finch song. Shown are representative examples of (A) simple stacked (SS), (B) simple noisy (SN), (C) complex stacked (CS) and (D) complex noisy (CN) syllables. Simple stacked syllables are represented by single harmonics stacks or clear tones, whereas simple noisy syllables are single elements that lack well defined harmonic structure. Complex stacked types contain a combination of harmonic stacks and noisy elements, whereas complex noisy have multiple noisy elements but no harmonic stacks. Right: Effects of alcohol on zebra finch song syllable types. Direction of arrows indicate direction of change (up or down), dashes indicate lack of change. Frequencies of directional shifts are tallied at the bottom (yellow).

**Figure 9:**
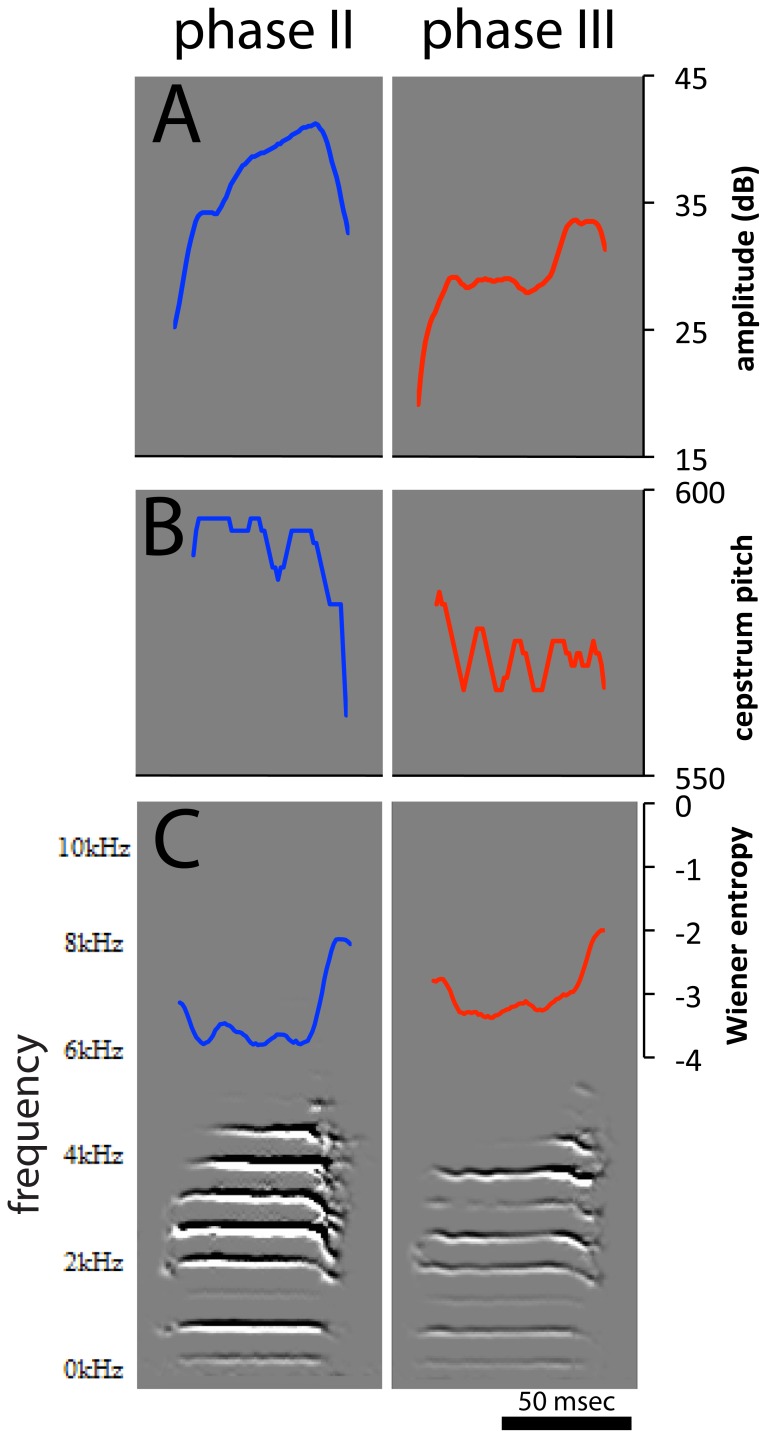
Effects of alcohol drinking on a specific zebra finch song syllable. Shown are spectrograms of a representative syllable recorded in Phases II (no alcohol; left) and III (alcohol, mean BEC = 53.4 mg/dl; right) overlaid with traces of (A) amplitude, (B) pitch, and (C) Wiener entropy (respective scales on the right). For this syllable there were significant decreases in mean amplitude and pitch, and an increase in entropy under alcohol; other acoustic features did not change (not shown).

We next wondered whether syllables of the same type showed consistent changes under alcohol. A distinctive quality of zebra finch song is the occurrence of harmonic stacks in some, but not all syllables. Depending on the presence of stacks, in combination with occurrence of a discrete temporal shift in acoustic structure, we categorized syllables into four types (representative examples in Fig. 8A–D), and recorded whether their acoustic parameters were affected by alcohol (a representative example of simple stack syllable analysis in Fig. 9). As summarized in Fig. 8E, syllables of the same type showed different effects under alcohol, thus syllable type did not predict the specific combination of spectral shifts under alcohol. We note, however, that except for duration, which could increase or decrease, the effects of alcohol on a given parameter were consistently in the same direction, even across syllable types, namely an increase in entropy and a decrease in all other acoustic parameters.

### Effects of alcohol on song stereotypy

The song of adult male zebra finches typically consists of bouts of repeated motifs with minimal differences across motif renditions. To test whether alcohol might affect song stereotypy, we examined whether motif % similarity, which measures the correlation between two songs assuming no shifts in syntax, was affected by alcohol. Because motif variability is low for motifs that occur early in a song bout [26], we separately analyzed motifs 1 and 3 to test whether alcohol has greater effects on motifs that occur late within song bouts. We did not find a significant effect of alcohol on similarity scores across bouts for motifs 1 (repeated measures ANOVA, F_1, 5_ = 1.08, p = 0.0678) or for motifs 3 (F_1, 5_ = 0.0001, p = 0.98). We also did not detect effects within song bouts between motifs one and three (F_1, 5_ = 0.06, p = 0.617). Values are presented in Table S3 in S1 File.

## Discussion

Here we found that when zebra finches drink alcohol, they can reach BECs comparable to those commonly seen in humans [27], [28], which measurably affects their song. The BECs achieved in our paradigm (Fig. 2) can be considered “risky” drinking based on the NIH definition for adult humans (between 50–80 mg/dl), with one bird approaching the NIH definition of binge drinking of >80 mg/dl [29]. Interestingly, when given alcohol finches showed increased fluid intake, indicating that alcohol-supplemented juice was not aversive. Consistent with moderate alcohol intake and absence of adverse effects like dehydration, we did not detect changes in the birds’ overall behavior or motor coordination. These observations indicate that drinking patterns and alcohol levels that are of relevance to human consumption have significant effects on the production of learned vocalizations in zebra finches. Thus, alcohol effects are distinct those of other factors known to affect song, such as age [30], social context [31] or stress [32]. Given the important parallels between birdsong and human speech and language, the zebra finch is potentially a powerful animal model system for understanding how alcohol affects a set of learned social behaviors that are highly relevant to humans.

Daily alcohol consumption in our paradigm did not correlate well with measured BECs. This is perhaps not surprising, given that the consumption measurements were integrated over the entire day, whereas the blood samples were taken at a single time. It is thus possible that the birds had highly variable drinking patterns, or that alcohol was very rapidly metabolized after ingestion. Compared to rodents [24], however, we found that finches clear alcohol quite slowly once high BEC levels are achieved, despite the relatively high general metabolic rates of birds [33]. These clearance data suggest that the variability in BECs is not due wide variations in ethanol metabolism in finches. While further analysis is required to better understand the relationship between voluntary intake and BEC levels, the free drinking paradigm we developed offers several advantages. Most importantly, significant increases in BECs can be achieved through an easy administration route that preserves normal zebra finch behaviors, allowing us to readily record song. Furthermore, we did not observe overt signs of stress with the free drinking paradigm. This was in sharp contrast to the injections for the clearance study, which were highly stressful and did not allow us to readily assess the effects of alcohol on song behavior. We also note that the use of a free drinking administration will be important for future analysis of the brain circuitry that are affected by alcohol. Previous studies in rodents, for example, have shown differences in brain activation patterns when given alcohol as an IP injection compared to self-administration [34], [35], possibly due to effects of fear associated with handling and unexpected intoxication.

Most birds did not appreciably adjust their singing rate or modify song stereotypy under alcohol, except for a small decline in the number of song bouts at the highest intake levels. The lack of a strong effect on these variables could be due to the fact that we evaluated only female-directed song, which differs from non-directed song and is under the control of different neuronal circuitry [36]. It is possible, for example, that significant stereotypy changes would require higher levels of intoxication or alternatively, song stability might be more sensitive to alcohol during undirected singing, which is less stereotyped [37]–[39]. Zebra finches exhibit ample song in the presence of either females or males [40], thus providing excellent opportunities to investigate how alcohol may affect activation of the song circuit during different social contexts.

The strongest effects of alcohol on song were on amplitude and entropy, detectable over whole motifs and at the individual syllable level. The effect on entropy, in particular, indicates a destabilizing effect of alcohol on song production, disrupting a bird’s ability to maintain its normal acoustic structure of song and its component syllables. Duration and several spectral features also showed measurable effects at the scale of certain syllables, but the extent and degree of change varied considerably across syllables and individuals, as opposed to the broad effects for amplitude and entropy seen over whole motifs. These effects were confirmed when analyzing how the magnitude of the acoustic changes were affected by increasing BEC values, with notable changes detected in a transition at around 40 mg/dl. Interestingly, this dose effect was not seen when examining the frequency of changes, as the fraction of syllables that showed a significant change did not correlate well with BEC levels. Overall, these observations suggest a possibly stochastic relationship between BEC levels and the number of acoustic features affected at the syllable level. Importantly, the different syllables of a typical finch song differ considerably in their spectral-temporal structure, reflecting diversity in the underlying neuronal encoding and/or modes of production, and some syllables likely require more complex encoding or production mechanisms. Based on the diverse response among syllables, we suggest that alcohol differentially affects the neuronal and/or neuromuscular mechanisms responsible for encoding specific syllables. We also note that the vocal learning process reflects the ability of a bird to modify its own vocalizations to match a model, or template. That ability implies that the vocal learning and production circuits are able to exert a fine control over the acoustic parameters of the learned vocalizations the bird produces, and we suggest that alcohol disrupts this control. Exploring these various effects through electrophysiological recordings of neuronal activity during singing under the influence of alcohol, would elucidate how neuronal firing patterns contribute to the acoustic structure of specific song syllables, besides addressing the mechanisms of alcohol action on vocalizations. Interestingly, although different syllables were not uniformly affected, the directions of change under alcohol were nearly always the same, resulting in a predictable effect for most acoustic features. The exception was duration, where syllables increased, decreased, or did not change. A comparative analysis of firing patterns across syllables may thus be particularly informative with regards to the neuronal encoding of the temporal structure of song elements.

Previous investigations of acute alcohol effects on human vocalizations include acoustic analyses of repeatedly occurring suprasegmentals (e.g. spectral features of individual syllables) such as pitch or fundamental frequency, formant tracking in individuals, changes in speaking frequency and duration, vocal intensity, and occurrence of non-fluencies. While even the best-controlled experiments cannot reliably identify a consistent spectral feature that changes with alcohol across all distinct elements of human speech [4], human inebriation generally increases fundamental frequency of speech elements [41]. Other factors such as the duration and intensity of phonation may also increase, and non-fluencies may occur, but mostly in severely intoxicated states. Despite this, the variability of acoustic features (e.g. fundamental frequency) has not been shown to change with intoxication [4], and not all subjects nor all elements of speech within an individual show a uniform response. For instance, 20% of subjects did not change their fundamental frequency despite dramatic changes in most (∼80%) individuals [41]. Thus, we conclude that alcohol effects on both human and songbird vocalizations are not uniform, but rather are specific to specific vocal elements both in extent and direction of change. Also importantly, acute alcohol exposure in humans does not strongly alter general peripheral motor function, as measured by isokinetic and isometric muscle performance, and creatine kinase levels [42], suggesting that the speech impairments of alcohol are more likely caused by acute effects on higher-level motor control pathways than on the neuromuscular system.

Alcohol may affect birdsong at several levels in the vocal control system (diagram in Fig. 1A). Beginning with pre-vocal motor areas the cortical-like song nucleus HVC contains individual neurons that project to vocal motor nucleus RA (HVC-_RA_ cells) and express sparse temporal coding by firing at discrete points during the production of a song motif. HVC-_RA_ neurons are important determinants of song temporal features, and based on our findings may also be an important target of alcohol on the timing aspects of song. RA output neurons, in turn, project to the medullary nXIIts nucleus that controls vocal (syringeal) and respiratory musculature and fires robustly throughout song duration. The firing patterns of these neurons could thus play important roles in mediating the effects of alcohol on the acoustic structure of most song syllable elements. Given the differential effects we observed, we predict that neuronal subsets within HVC and/or RA are differentially susceptible to alcohol, but we also note that possible effects at the level of the neuromuscular junction or syringeal musculature cannot be discarded.

Overall, alcohol has clear effects on zebra finch song, establishing this species as an informative model to study the effects of alcohol on a cognitive skill with similarities to human speech acquisition. Because the song control circuitry is well mapped and the neuronal activity of its elements can be readily accessed by various methods, including analysis of activity-dependent immediate early genes, and *in vivo* electrophysiological recordings in freely singing birds, specific hypotheses about how alcohol affects the neuronal control of learned vocalizations are highly testable. Such advances would help elucidate how alcohol affects vocal motor control in humans, where speech is markedly affected through as yet unclear mechanisms. An intriguing potential application would be the use of bioacoustics analysis of vocalizations to reliably detect inebriation or even mild intoxication in humans.

## Methods

### Study Animals

Adult male zebra were obtained from a commercial breeder or were born in our aviary and were at least 120 days old at the time of the experiments. Finches were housed 12D/L conditions in single-sex group cages in our institutionally managed aviary prior to the onset of the experiments, and brought to the lab for the behavioral experiments described in this paper. All research was approved by the Oregon Health and Science University Institutional Animal Care and Use Committee (protocol no. IS2313).

### Experimental Design

We housed zebra finches in sound isolation chambers, equipped with a duplex cage with perches, food, and drink. With longitudinal sampling we measured changes in song in individual finches over several days of recording, as we changed the contents of their drinks (Fig. 1B). Initially we presented birds with water in a single bottle and recorded song for 1–2 days to establish baseline conditions (Phase I). We then switched the drinking solution to 50% white grape juice and recorded song for 1–2 days (Phase II), to control for the possibility that juice alone had an effect, We then added 6.5% ethanol to the juice and sampled song over 3–4 days of drinking (alcohol group, Phase III); we also recorded song from a parallel set of birds that continued to be exposed to juice only for a similar number of days (control group, Phase III). Since the finches freely consumed the drinking solution the degree of intoxication was dependent on the drinking characteristics of each bird. Thus, alcohol consumption was not directly forced, but a fresh water alternative was not provided. We monitored birds closely for changes in weight or other signs of dehydration such as stupor or changes in tissue turgor, for general behaviors including feeding, preening, perching, flying inside the cage and calling, as well as stress related behaviors such as posture, puffed feathers, inactivity or non-responsiveness towards other birds.

To study the metabolic clearance of alcohol, separate groups of finches were administered two different doses, 2.0 g/kg (n = 4 birds) or 3 g/kg (n = 3) of alcohol (delivered as 20% in PBS; 150–240 µl injection volume). In order to approximate the paradigm used for rodents, we attempted an intraperitoneal (IP) administration, consisting of injections ∼5 mm above the cloacal opening along the midline, taking care to avoid the liver, which is visible through the skin. We note the absence of cackling with breathing, which would occur if we injected into the birds’ pulmonary air sac system, and that the initial BEC measurements had low variability, indicating that the injections were all correctly placed. We then replaced the birds individually in a quiet cage. At 30, 60, 90, 150 or 210 min following the alcohol administration, we lanced the brachial vein with a disposable needle and drew a ∼50 µl blood sample into a heparinized capillary tube. Serum was isolated by centrifugation in a microcentrifuge for 3 min and analyzed with an Analox GL5 analyzer calibrated to a known standard. Injected birds showed rapid changes in posture and quickly entered an intoxicated stupor marked by puffed feathers, closed eyes and general inactivity. At the 3 g/kg dose, birds did not fly or perch, but sat on the cage floor with their eyes closed. If handled, birds would briefly open their eyes; 30–60 minutes into the experiment birds were capable of moving about the floor of the cage, but did not perch.

### Recording

In the presence of females, male zebra finches reliably produce female-directed song as part of their courtship behavior [25]. Cages were set up for singing males to perch and sing towards a female, and into a microphone at the opposite end of the female’s cage. Cages were fit into acoustically isolated boxes supplied with fresh air and broad spectrum LED lights on a 12D/L timer. We used Audix TR40 microphones connected to an SM Pro Audio PR8-MK2 preamplifier, fed into a microcomputer with an Aardvark Direct Pro LX6 sound card running Sound Analysis Pro software. Singing was recorded and processed with the settings for zebra finch song. Four hrs after lights on, a female was added to the adjacent cage for a 2 hr recording session. At the termination of each session, the female was removed, males were weighed, and a blood sample was collected for measurement of BEC; sample processing was as above for the alcohol metabolic clearance study. The amount of liquid consumed over the previous 24 hrs was also measured. Thus, fluid intakes are reported over 24 hour periods and the BECs were point measurements made at the termination of each recording session. We then replenished food and drink, according to their treatment.

### Analysis

To address whether alcohol affects song, we analyzed rates of song production, song acoustic features and song stereotypy. For the song production analysis, we tallied the number of songs produced during 2 hrs of female exposure, and calculated the mean number of motifs in each song, and the number of lead notes that preceded each song.

Acoustic features of song were acquired with the Explore and Score feature of SAP for all full motifs that were produced during the two hrs of female presence, excluding background noise or artifacts. We analyzed motif duration, amplitude, pitch, frequency modulation (FM), amplitude modulation (AM) and Wiener entropy. Duration was determined manually and blind to Phase as the interval between the beginning of the first syllable and the end of the last syllable of the motif, and the other acoustic features were based on mean values within this interval. We imposed a smoothed amplitude-based segmentation rule over all recordings so that measurements did not include silent gaps between syllables. Amplitude calculated by SAP is the absolute deviation of the vocal signal from the baseline. Pitch is broadly defined as the oscillation frequency of sound, and is calculated from SAP’s cepstrum pitch, which is a combination measurement of the fundamental frequency of oscillation for harmonic sounds, and the mean frequency of oscillation for sinusoidal (clear) or poorly defined frequencies. FM is the change of sound frequencies with time and AM is the time derivative in amplitude across all frequencies. Wiener entropy is the width and uniformity of the power spectrum. The last four features are largely independent of the amplitude of sound [19] and do not depend on the proximity of the bird to the microphone.

The motif-scale analysis was followed by a syllable-level analysis for all birds in the alcohol group, where all syllables within a motif (2–6 per bird) that were recorded on the day of the highest BEC obtained for that individual (Phase III) were compared to the same syllables recorded before the alcohol exposure (Phase II). We first segmented the sounds based on smoothed amplitude to identify putative syllables. Next we calculated the mean spectral features for each of the segmented sounds, and used these spectral features in a nearest-neighbor clustering algorithm (in SAP) to classify individual syllables. Sounds that did not cluster, such as cage noise and non-song vocalizations, were discarded. The clustering quality was confirmed by visual examination of syllable assignments within the spectrograms to ensure correct cluster assignments for all syllables. We note that cluster assignment was consistent across days and not affected if the bird had alcohol in its system. In two cases SAP could not reliably separate syllables within the birds’ motifs, likely due to a flat spectral quality of these songs. Thus, we assigned syllables manually for these birds. Next we compared the values between the corresponding syllables recorded under alcohol (Phase III) and prior to exposure (Phase II).

We next categorized syllables into one of four groups based on their visual appearance in the spectrogram, as in [43]: (1) simple stacked syllables, represented by only harmonic stacks or clear tones, (2) simple noisy syllables, represented by sounds that lacked harmonic structure, but had no temporal variation, (3) complex stacked syllables, illustrated by those syllables that contained both a harmonic stack, and a component whose spectral structure varied temporally, and (4) complex noisy syllables, whose spectral structure varied temporally, but which lacked a clear harmonic component. We compared for each syllable type the acoustic features recorded under alcohol (Phase III) vs prior to exposure (Phase II).

For the stereotypy analysis, we tested the effect of alcohol with single motif libraries of 16-bit.WAV files (10–20 high quality randomly selected motifs that lacked background cage noises or female calls) obtained both prior to alcohol exposure (Phase II) and on the day of highest measured BEC for each bird in the study (Phase III). We analyzed the first and third motifs in each song bout separately, as previous reports suggest these differ in their degree of stereotypy across renditions [26]. Not all birds in our study produced sufficient numbers of bouts with greater than three motifs, so we did not examine stereotypy that occurred later in songs, as in [26]. To calculate % stereotypy scores for each motif, we performed all possible pairwise comparisons of all renditions of the same motif, with the SAP batch feature [19] and recorded the resulting means. We then compared values obtained for each motif across experimental Phases (Table S3 in S1 File, Motifs 1 and 3). To generate a “Within Bout” average % similarity score, we next performed pairwise comparisons of the first and third motifs from all song bouts. We then compared the resulting values across experimental Phases (Table S3 in S1 File, “Within Bout”).

To analyze these longitudinal data we used MANOVA and repeated measures models, followed by *post hoc* clustering with principle components analysis (PCA).

We used separate MANOVAs to look for changes in any of the six response variables when we contrasted the different experimental Phases. We calculated zero-centered residuals of whole-motif amplitude and entropy around the mean values of individual finches for each BEC measurement. We also tested acoustic features measured at the syllable level with t-tests, adjusting for multiple comparisons with a Bonferroni correction to α, followed by comparison of Phase III values at each day to the Phase II value using Dunnett’s Method. We also tested the effects of BEC on the (1) magnitude of the acoustic features and on (2) the fraction of acoustic features that changed (number of acoustic features that changed relative to the total number of features analyzed) for each syllable that each bird produced. For the latter two comparisons we used separate general linear models for each feature with BEC as the main effect and individual bird and syllable nested within individual bird as random effects. Data, once extracted from databases created with SAP, were stored and managed in Microsoft Excel and statistical tests were performed with JMP Version 10.

## Supporting Information

S1 Fig **Total fluid intake across phases of the experiment.** Plotted are mean daily fluid intake values of zebra finches under the experimental paradigm shown in Fig. 1B. Error bars are standard errors of the means. * indicates a significant difference.
(TIF)

S2 Fig **The relationship between BEC following IP injections of 2 and 3 g/kg alcohol.** Symbols connected by black lines are finches and grey lines are C57 mice, from [24].
(TIF)

S3 Fig **Effect of BEC on mean number of song bouts per hour.** Black symbols/line show the decline in song rate of most birds in the alcohol group (p = 0.0084, R^2^ = 0.29), while one individual (in orange) shows exceptionally high singing rates during Phase III.
(TIF)

S4 Fig **Dose-relationships between BEC and syllable-level (A) amplitude and (B) duration, and (C) entropy.** The vertical axes are residual values centered around syllable and individual bird means. Individual birds are represented by unique colors; the traces are spline fits (λ = 100,000).
(TIF)

S1 File **Combined supporting tables of alcohol effects singing parameters.** Table S1 documents statistical effects of alcohol on the amount of song, Table S2 documents statistical effects of alcohol on song acoustic features and Table S3 lists the effect of alcohol on song stereotypy.
(DOCX)

S2 File **Video of alcohol effects on the acoustic features of select zebra finch syllables.** Syllables include, first, the example from Fig. 9, and second, a syllable with higher spectro-temporal complexity. Vocalizations are shown serially, first while drinking juice and then while drinking 6.5% alcohol in juice, at normal and reduced speeds.
(MP4)
