# Supplementary figures and images for: Drinking Songs: Alcohol Effects on Learned Song of Zebra Finches
Source: PLoS One. 2014 Dec 23;9(12):e115427. doi: 10.1371/journal.pone.0115427 (PMC4275239; doi:10.1371/journal.pone.0115427)

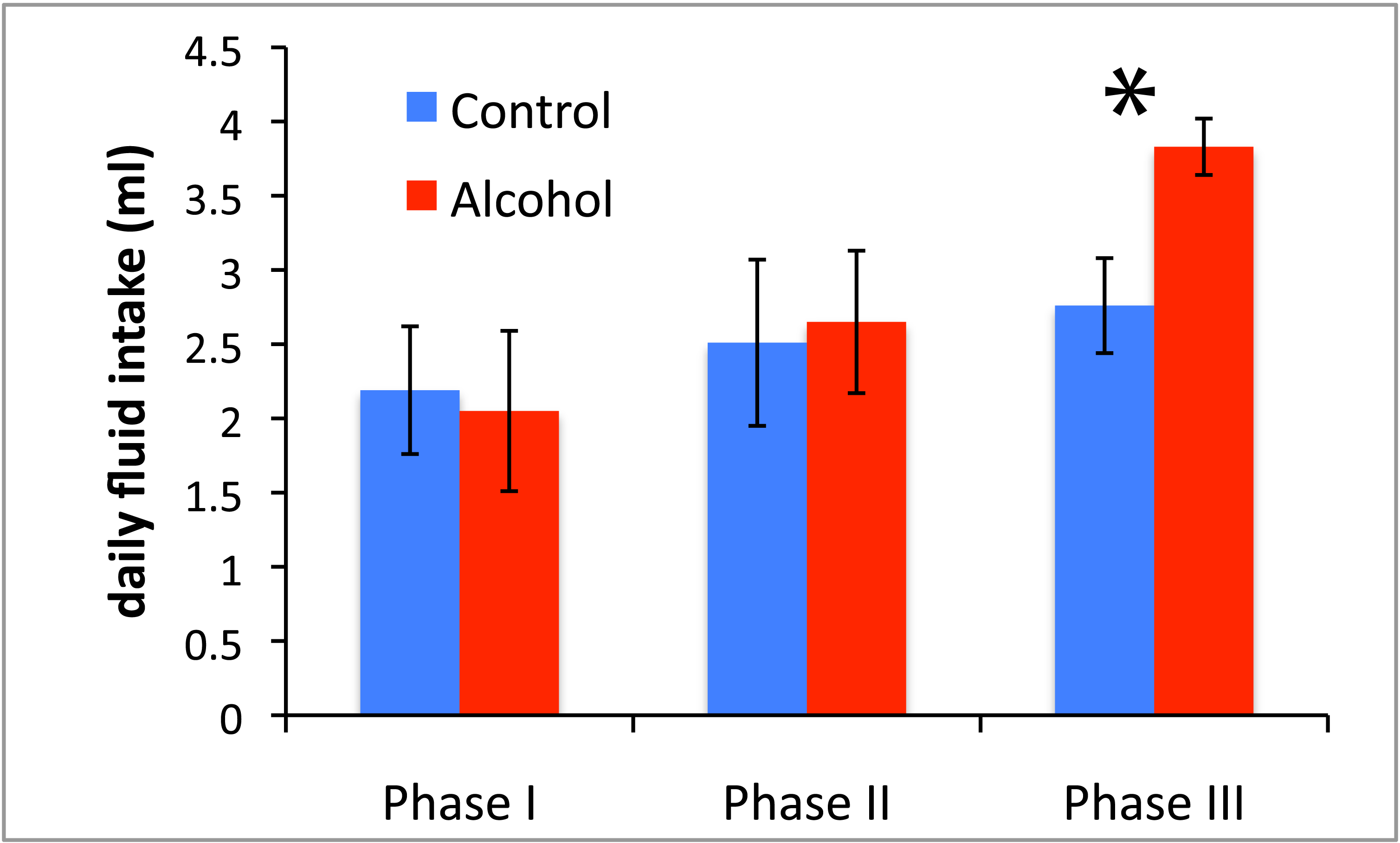

Supplement: S1 Fig — Total fluid intake across phases of the experiment. Plotted are mean daily fluid intake values of zebra finches under the experimental paradigm shown in Fig. 1B. Error bars are standard errors of the means. * indicates a significant difference. (TIF) [file pone.0115427.s001.tif]

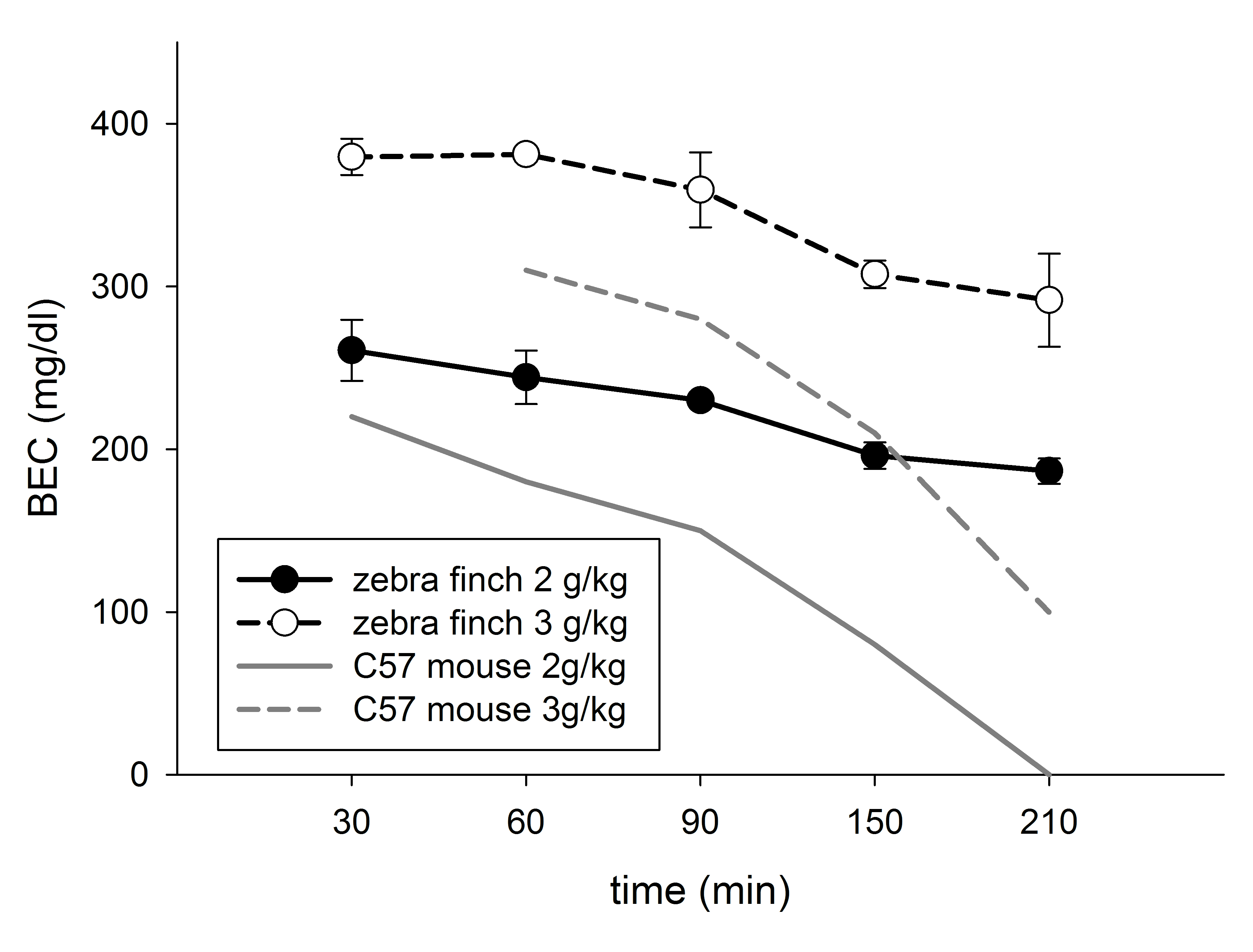

Supplement: S2 Fig — The relationship between BEC following IP injections of 2 and 3 g/kg alcohol. Symbols connected by black lines are finches and grey lines are C57 mice, from [24]. (TIF) [file pone.0115427.s002.tif]

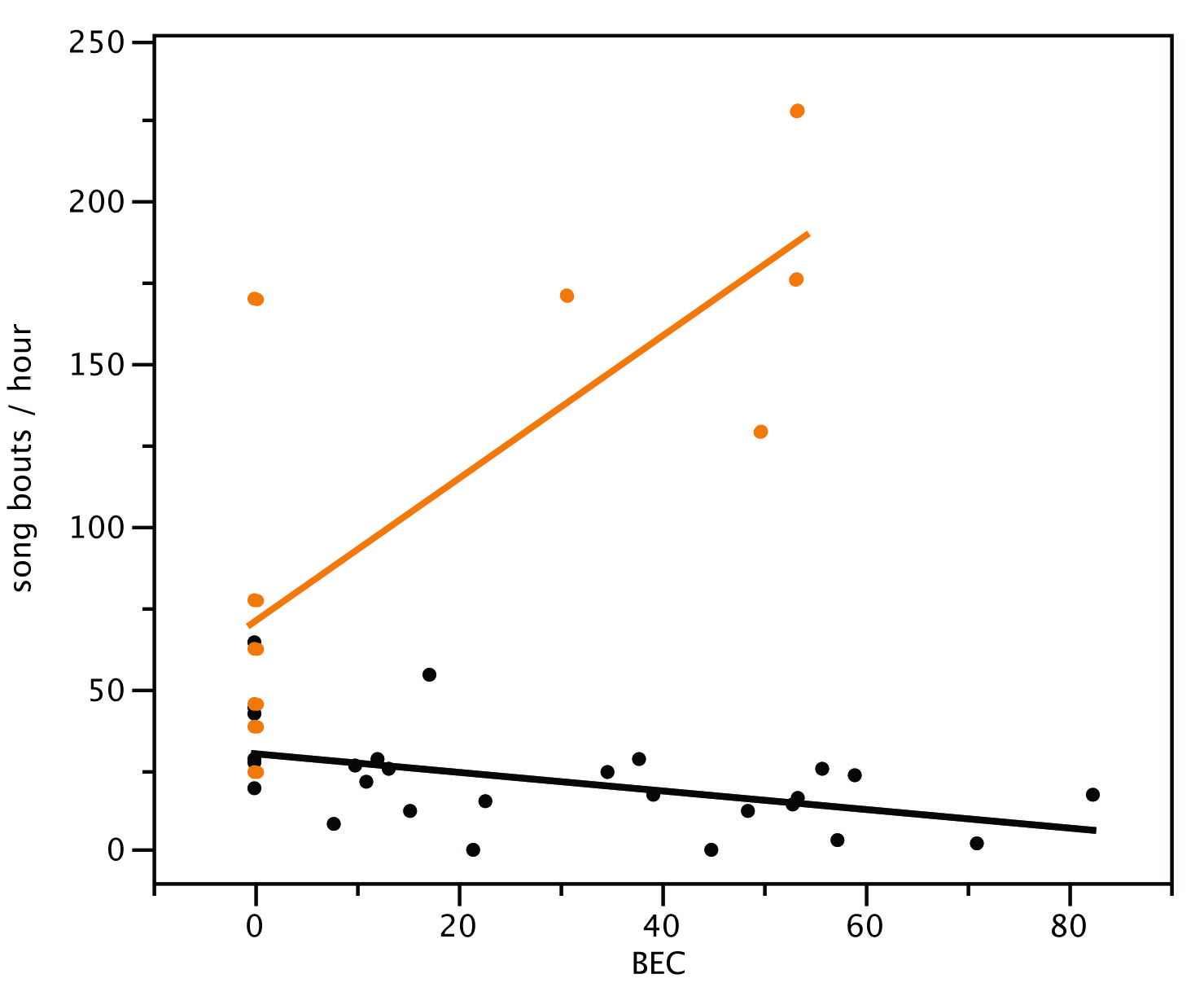

Supplement: S3 Fig — Effect of BEC on mean number of song bouts per hour. Black symbols/line show the decline in song rate of most birds in the alcohol group (p = 0.0084, R2 = 0.29), while one individual (in orange) shows exceptionally high singing rates during Phase III. (TIF) [file pone.0115427.s003.tif]

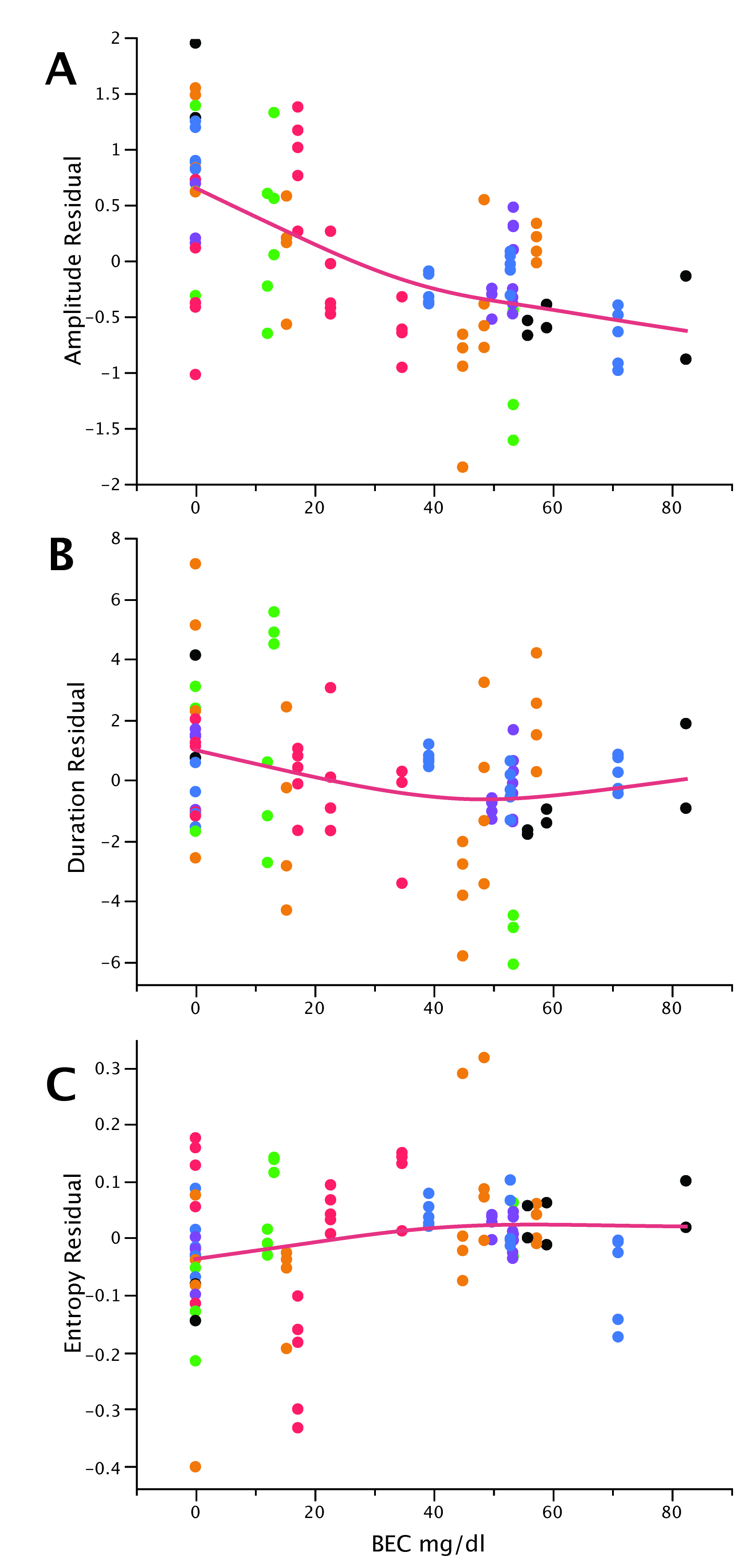

Supplement: S4 Fig — Dose-relationships between BEC and syllable-level (A) amplitude and (B) duration, and (C) entropy. The vertical axes are residual values centered around syllable and individual bird means. Individual birds are represented by unique colors; the traces are spline fits (λ = 100,000). (TIF) [file pone.0115427.s004.tif]
