# Supplementary material for: Drinking Songs: Alcohol Effects on Learned Song of Zebra Finches
Source: PLoS One. 2014 Dec 23;9(12):e115427. doi: 10.1371/journal.pone.0115427 (PMC4275239; doi:10.1371/journal.pone.0115427)
Supplement: S1 File — Combined supporting tables of alcohol effects singing parameters. Table S1 documents statistical effects of alcohol on the amount of song, Table S2 documents statistical effects of alcohol on song acoustic features and Table S3 lists the effect of alcohol on song stereotypy. (DOCX) [file pone.0115427.s005.docx]

**FILE S1: Supplemental Tables**

Table S1. Effects of alcohol on the amount of singing. Repeated measures ANOVA compares the number of lead notes and motifs within a bout and the number of singing bouts per hour across groups. Phase is the repeated variable that transitions from juice (Phase II) to ethanol (Phase III), compared between the group that received alcohol in Phase III vs. controls, which did not. * indicates a significant difference; ^#^ minus the two birds that show a large increase later in the trials

| **Parameters** | **Variables** | **F** | **d.f.** | **p** |
| --- | --- | --- | --- | --- |
| leads/bout | Group | 0.000414 | 1, 9 | 0.9527 |
|  | Phase | 1.13 | 2, 8 | 0.0487* |
|  | Interaction | 0.123 | 2, 8 | 0.6292 |
|  |  |  |  |  |
| motifs/bout | Group | 0.0222 | 1, 10 | 0.6476 |
|  | Phase | 0.187 | 2, 9 | 0.4619 |
|  | Interaction | 0.197 | 2, 9 | 0.4451 |
|  |  |  |  |  |
| bouts/hr | Group | 0.00395 | 1, 10 | 0.8464 |
|  | Phase | 0.349 | 2, 9 | 0.26 |
|  | Interaction | 0.031 | 2, 9 | 0.8731 |
|  |  |  |  |  |
| bouts/hr ^#^ | Group | 0.00722 | 1, 8 | 0.8162 |
|  | Phase | 0.306 | 2, 7 | 0.3928 |
|  | Interaction | 0.535 | 2, 7 | 0.2233 |

Table S2. Effects of alcohol on song spectral features. Post-hoc t-tests were run separately for the control and alcohol groups. * indicates significant differences; we note that for all parameters in both groups there were significant bird ID effects, reflecting significant individual variability.

| **Spectral Feature** | **Effect** |  | **control group** | | **alcohol group** | |
| --- | --- | --- | --- | --- | --- | --- |
|  |  | d.f. | F | p | F | p |
| Amplitude | Phase II-III | 1, 5 | 1.03 | 0.3200 | 15.3963 | **0.0007*** |
|  | Bird ID | 5, 5 | 323.40 | <0.0001 | 181.97 | <0.0001 |
| duration | Phase II-III | 1, 5 | 0.0027 | 0.9594 | 0.1731 | **0.6814** |
|  | bird ID | 5, 5 | 5104.843 | <.0001 | 4087.13 | <.0001 |
| pitch | Phase II-III | 1, 5 | 3.9225 | 0.0592 | 3.1133 | **0.0915** |
|  | bird ID | 5, 5 | 491.7016 | <.0001 | 413.04 | <.0001 |
| FM | Phase II-III | 1, 5 | 2.8928 | 0.1019 | 0.9450 | **0.3416** |
|  | bird ID | 5, 5 | 193.5141 | <.0001 | 142.2554 | <.0001 |
| AM | Phase II-III | 1, 5 | 1.7505 | 0.1983 | 3.2593 | **0.0847** |
|  | bird ID | 5, 5 | 22.4337 | <.0001 | 94.1853 | <.0001 |
| entropy | Phase II-III | 1, 5 | 2.2798 | 0.1441 | 8.1834 | **0.0091*** |
|  | bird ID | 5, 5 | 16.8364 | <.0001 | 92.2445 | <.0001 |

Table S3. Effects of alcohol on song stereotypy. Mean % similarity scores for across bout and within bout comparisons prior to and during alcohol. Values are mean ± s.d.

|  | **Motif 1** | **Motif 3** | **Within bout** |
| --- | --- | --- | --- |
| Phase II | 97.5±1.1 | 96.0±2.6 | 95.5±3.8 |
| Phase III | 96.6±1.5 | 96.0±2.7 | 96.0±3.1 |
